# Sociodemographic inequalities in the incidence of COVID-19 in National Household Sample Survey cohort, Brazil, 2020

**DOI:** 10.1590/1980-549720240012

**Published:** 2024-03-18

**Authors:** Italo Wesley Oliveira Aguiar, Elzo Pereira Pinto, Carl Kendall, Ligia Regina Franco Sansigolo Kerr

**Affiliations:** I Universidade Federal do Ceará, Postgraduate Program in Public Health – Fortaleza (CE), Brazil.; II Fundação Oswaldo Cruz, Instituto Gonçalo Moniz, Center for Data and Knowledge Integration for Health – Salvador (BA), Brazil.; III Tulane University School of Public Health and Tropical Medicine – New Orleans, Louisiana, USA.

**Keywords:** Sociodemographic factors, COVID-19, Demographic surveys, Cohort study, Survival analysis, Fatores sociodemográficos, COVID-19, Inquéritos populacionais, Estudo de coorte, Análise de sobrevivência

## Abstract

**Objective:**

To verify the association between sociodemographic factors and the time until the occurrence of new cases of COVID-19 and positive tests for SARS-CoV-2 in Brazil, during the period from May to November 2020, based on a cohort of Brazilians participating in the COVID-19 National Household Sample Survey.

**Methods:**

A concurrent and closed cohort was created using monthly data from the PNAD COVID-19, carried out via telephone survey. A new case was defined based on the report of the occurrence of a flu-like syndrome, associated with loss of smell or taste; and positivity was defined based on the report of a positive test, among those who reported having been tested. Cox regression models were applied to verify associations. The analyzes took into account sample weighting, calibrated for age, gender and education distribution.

**Results:**

The cumulative incidence of cases in the overall fixed cohort was 2.4%, while that of positive tests in the fixed tested cohort was 27.1%. Higher incidences were observed in the North region, in females, in residents of urban areas and in individuals with black skin color. New positive tests occurred more frequently in individuals with less education and healthcare workers.

**Conclusion:**

The importance of prospective national surveys is highlighted, contributing to detailed analyzes of social inequalities in reports focused on public health policies.

## RESUMO

**Objetivo:**

Verificar a associação entre fatores sociodemográficos e o tempo até a ocorrência de novos casos de COVID-19 e de testes positivos para Sars-CoV-2 no Brasil, durante o período de maio a novembro de 2020, com base em uma coorte dos brasileiros participantes da Pesquisa Nacional por Amostra de Domicílios COVID-19.

**Métodos:**

Foi constituída uma coorte concorrente e fechada utilizando dados mensais da Pnad COVID-19, realizada por inquérito telefônico. Um caso novo foi definido com base no relato da ocorrência de um quadro de síndrome gripal, associado à perda de olfato ou paladar; e a positividade foi definida com base no relato de um teste positivo, entre os que referiram ter sido testados. Foram aplicados modelos de regressão de Cox para verificar associações, considerando a ponderação amostral, calibrada para a distribuição etária, de sexos e de escolaridade.

**Resultados:**

A incidência acumulada de casos na coorte fixa geral foi de 2,4%, enquanto a de testes positivos na coorte fixa testada foi de 27,1%. Verificou-se maiores riscos nas regiões Centro-Oeste, Norte e Nordeste, entre mulheres, residentes em áreas urbanas, pessoas com escolaridade até o ensino médio, com a cor da pele declarada como preta e trabalhadores da área da saúde. Indivíduos com menor escolaridade e profissionais de saúde apresentaram maior frequência de novos testes positivos. Novos testes positivos ocorreram com maior frequência em indivíduos com menor escolaridade e trabalhadores da área da saúde.

**Conclusão:**

Foram observados riscos desiguais entre os estratos populacionais comparados. Destaca-se a importância da realização de inquéritos nacionais prospectivos na investigação de iniquidades em saúde.

## INTRODUCTION

Monitoring a nation's sociodemographic and health indicators is essential to identify social inequalities and to analyze whether the State is protecting the population's rights and interests. Among the surveys on the Brazilian population, the Continuous National Household Sample Survey (*Pesquisa Nacional por Amostra de Domicílios* – Pnad), organized by the Brazilian Institute of Geography and Statistics (*Instituto Brasileiro de Geografia e Estatística* – IBGE), stands out^1^.

In 2020, during the first months of the COVID-19 pandemic, the socioeconomic situation of the Brazilian population was suddenly shaken, reinforcing the need to understand the impact suffered on income distribution and social structure. That year, IBGE used the Continuous Pnad methodology to develop Pnad COVID-19, with the additional objective of estimating the number of people with reported symptoms associated with flu syndrome^2^, adding an important component to health surveillance^3^.

The results of Pnad COVID-19 were published in monthly editions from May to November 2020^4^, as a series of cross-sectional surveys. However, one of the most notable characteristics of this research is its fixed sample, whose households interviewed in the first month of data collect remain in the sample in subsequent months^2^. The use of techniques to connect records of individualized Pnad COVID-19 data allows increasing the capacity of data obtained in cross-sectional studies, when transformed into a nationwide prospective cohort study^1^.

In the global context, the application of cohort studies is of great importance to generate knowledge about the pandemic. In the United Kingdom, for example, in a cohort study of residents across the country, a higher risk of infections was found in males and with a lower level of education^5^. In Denmark, a national occupational cohort allowed identifying a higher risk in healthcare occupations^6^.

In Brazilian territory, information on the epidemiological situation of COVID-19 was obtained through cross-sectional serological surveys^7^, ecological studies based on data from health information systems^8^ and through internet-based surveys^9^. Despite the relevance of these designs, there is a lack of national research that considers the temporality between exposure and outcome from an individual and prospective point of view, which has not been carried out exclusively with individuals who sought health services or with data from virtual questionnaires.

This work aimed to verify the association between sociodemographic factors and the time until the occurrence of new cases of COVID-19 and positive tests for Sars-CoV-2 in Brazil, from May to November 2020, based on a cohort of Brazilians participating in Pnad COVID-19.

## METHODS

### Study design

A concurrent, closed, passive participation cohort was constructed, using sociodemographic and clinical-epidemiological information. Data referred to Brazil as a whole, between May and November 2020, and came from the series of population-based telephone surveys of Pnad COVID-19. Interviews were carried out in a fixed sample of households, which allowed records to be linked by identifying key variables that distinguish participants from each edition^2^.

### Background

This research referred to the entire national territory, with monthly interviews carried out between May and December 2020, referring to the seven months following the two to three months after the start of the COVID-19 pandemic in Brazil. Territories comprising indigenous villages, barracks, military bases, accommodation, camps, vessels, boats, ships, penitentiaries, penal colonies, prisons, jails, nursing homes, orphanages, convents, hospitals, and settlement project farm villages were excluded from the coverage area, in addition to census tracts located on indigenous lands^10^.

### Participants

Target population comprised people residing in permanent private homes in the research area. Pnad COVID-19 data collect began on May 4^th^, 2020, with interviews carried out by telephone in approximately 48 thousand households per week, totaling approximately 193 thousand households per month throughout the national territory^2^.

The fixed sample of the series of surveys was based on the Continuous Pnad sample from the 1^st^ quarter of 2019, which included around 211 thousand households. Cluster sampling technique was used in two stages of selection, with stratification of primary sampling units (PSU). In the first stage, PUS were selected with a probability proportional to the number of households in each defined stratum. In the second stage, 14 permanent private households occupied in each PSU in the sample were selected, by simple random sampling from the National Register of Addresses for Statistical Purposes (*Cadastro Nacional de Endereços para Fins Estatísticos* – Cnefe)^1^. The adaptation of Continuous Pnad into a telephone survey required IBGE to carry out a pairing between telephone operator databases and administrative records to obtain telephone numbers, landline or mobile, of individuals who were surveyed in the 1^st^ quarter of 2019, which resulted in a match of 92% of the desired sample^11^.

**Figure 1:**
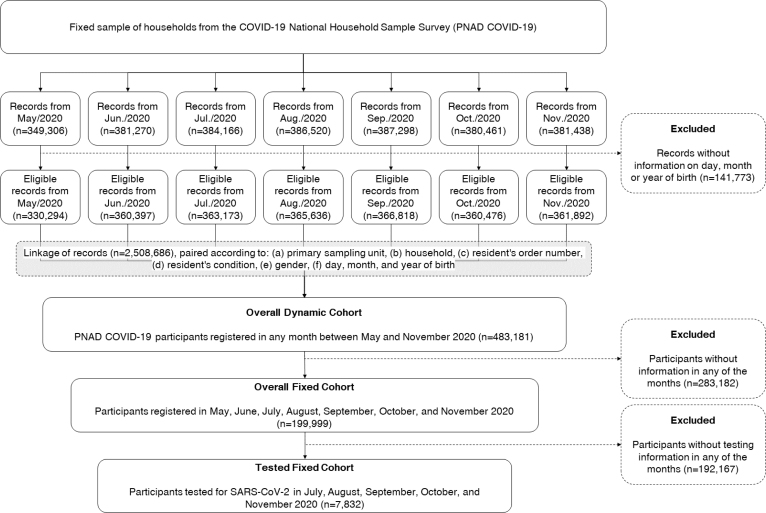
Sample selection and constitution of the overall dynamic, fixed, and tested fixed cohorts, derived from the Pnad COVID-19 editions. Brazil, May-November/2020.

Based on individual records, inclusion criteria were established to create three cohorts for this study: a general dynamic cohort and two fixed cohorts derived from it, one general and the other tested (Figure 1). Overall dynamic cohort was made up of any participants linked between the months of the survey, regardless of the number of records. Overall fixed cohort was formed by a subcohort of the general dynamic cohort, covering only individuals registered in all interviews. The fixed cohort tested consisted only of those individuals from the general fixed cohort who had been tested on all occasions.

Insufficient information on day, month, and year of birth were considered exclusion criteria for all cohorts, since these variables were part of the key used to link records collected at different times. The percentage of interviews excluded by this criterion was equivalent to 5.3% of all records carried out between May and November 2020.

### Variables

Interviews were structured according to the official Pnad COVID-19^12^ questionnaire, which was divided into two parts: one focused on health issues, specifically on self-reported symptoms associated with flu syndrome and testing for Sars-CoV-2; and another addressing work issues.

The variables used in this study were categorized according to their purpose, being considered as: variables that constitute the record linking key and descriptive variables of the sample. The record linking key is a set of variables used to identify the same individual throughout the Pnad COVID-19 editions. This combination was consulted in the work of Teixeira Júnior et al.^13^, who linked the quarters of Continuous Pnad between 2017 and 2018, and in the study by Monteiro^14^, who dealt with the quarters from 2012 to 2017. The variables are:

- Primary sampling unit (*psu*) – Identification of the set of census tracts that, when aggregated, represent area units with a minimum size for research. Each PSU is limited to its reference municipality, not exceeding municipal territorial limits.
- Household selection number (*v1008*) – Identification of one of the 14 households that were randomly selected in each primary sampling unit. Categories: Numbers 1 to 14.
- Condition of the resident in the household (*a001a*) – The residents of the household were listed, and the interviewer indicated the person responsible for the household. Next, the relationship between residents and the head of the household was questioned. Categories: Head of the household (1); Spouse or partner of different gender (2); Spouse or partner of the same gender (3); Child of the head of the household and spouse (4); Child only of the head of the household (5); Child only of the spouse (6); Son-in-law or daughter-in-law (7); Father, mother, stepfather or stepmother (8); Father-in-law (9); Grandchild (10); Great-grandchild (11); Sibling (12); Grandfather or grandmother (13); Another relative (14); Nonfamily – Nonrelative who does not share expenses (15); Cohabitant – Nonrelative who shares expenses (16); Pensioner (17); Domestic worker (18); Relative of the domestic worker (19).
- Resident order number *(a001*) – After defining the relationship between each resident and the head of the household, the IBGE interview system assigned a sequential order number to each individual. Categories: Numbers from 1 to 30.
- Gender (*a003*) – Categories: Male (1); Female (2).
- Day, month, and year of birth (*a001b1*, *a001b2*, *a001b3*) – Categories: Numbers from 1 to 31 for the day, from 1 to 12 for the month, and from 1890 to 2020 for the year.

The juxtaposition of these variables formed the unique individual key for each participant. For example, in a hypothetical situation: a person located in the census sector included by PSU with identification "230022987", in the randomly drawn household number "6", with order number "5", being the son of the couple ("4"), of male gender ("1"), and who was born on May 11^th^ ("11") ("5"), 1995 ("1995") would receive the unique key value "23002298765411151995", which remained the same for that individual in all editions of Pnad COVID-19.

In addition to the variables used in the identification key, some variables were selected to describe the sample based on the complete set of variables from Pnad COVID-19^12^. Below, these variables are listed, with their categories:

- Region (Central West; Northeast; North; Southeast; South).
- Age range, in completed years (0–9; 10–19; 20–29; 30–39; 40–49; 50–59; 60–69; 70–79; ≥80). Note: this variable was renamed and recategorized exclusively for the construction of the graphics, now being called "Stage of life" (Child, 0-9 years old; Adolescent, 10-19 years old; Young adult, 20-39 years old; Middle aged, 40-59 years; Aged, 60 years old or older);
- Gender (Female; Male).
- Race or skin color (White; Black; Yellow; Brown; Indigenous; Not declared).
- Area of residence (Urban; Rural).
- Education of people aged 25 years old or older (Incomplete primary education or less; Complete primary education; Completed secondary education; Completed higher education or more; Not applicable);
- Work of people aged 14 years old or older (Health; Transportation; Food; Other higher education professions; Commerce; Industry; Agriculture; Other services; Not declared; Not applicable).
- Self-reported case of COVID-19 in the previous week (Yes; No). It was the outcome investigated in the overall fixed cohort, inspired by the Brazilian Ministry of Health's confirmed clinical case definition criteria^15^. "Yes" was considered to be individuals with an acute onset of loss of smell or taste, along with a flu-like syndrome, defined as at least two of the following signs or symptoms: headache, runny nose, cough, sore throat, fever, loss of smell or taste and gastrointestinal symptoms;
- Self-reported Sars-CoV-2 positivity (Yes; No). The outcome for the fixed cohort tested was considered, derived from the answers to the question about performance, type, and result of a test for positivity for Sars-CoV-2 (oral or nasal swab; finger prick; or venipuncture).

### Bias

The cohorts were formed by individuals living in permanent private households who responded to all interviews, carried out by telephone. Thus, it is important to consider the possibility that age^16^ and gender^17^ profiles differ in their availability to respond to questionnaires during business hours, as well as the fact that education profile causes differences between those most likely to participate in the research^18^ and those who have of active phone numbers^19^. To reduce these potential biases, the sample was subjected to weighting and post-stratification techniques, which are described below.

### Statistical analysis

The microdata from the Pnad COVID-19 editions of May, June, July, August, September, October, and November were accessed through the IBGE electronic address^20^, in June 2023.

After selecting the overall fixed cohort, the original sampling weights were adjusted for this subsample, aiming to account for discrepancies between the cohort and the population. To this end, population distributions according to age range, gender, and education were estimated for May 2020, based on weighted Pnad counts. These distributions were used in the post-stratification process of sample weights, according to the iterative proportional adjustment method, or raking^21^. After adjusting weighting, the overall fixed cohort began to better represent the distribution of age range, gender, and education of the Brazilian population, and the fixed cohort tested began to refer to the Brazilian subpopulation that underwent testing on a monthly basis between July and November of 2020.

In the descriptive analysis, the absolute frequency, the weighted relative frequency, and the respective 95% confidence intervals (95%CI) were described. Symptom prevalence and proportions of positive tests were presented in bar graphics. Incidence density resulted from the division between the estimated number of first events and the number of months contributed by individuals in each group (person-month), multiplied by a thousand.

Statistical significance of the association with the time until the first occurrence of new cases of COVID-19 and new positive tests for Sars-CoV-2 was evaluated in a Cox regression model, using the Breslow method to deal with ties. The assumption of proportional hazard rates was verified by graphical analysis, in which the accumulated probability of the events occurring was estimated based on the non-parametric weighted Kaplan-Meier statistics.

Alpha significance level was set at 5% (p<0.05). All data were processed, stored, and analyzed using the statistical software Stata/MP, version 17, including pairing, weighting, and post-stratification of the sample. The survey module was used to take into account the complex sampling design of the survey.

### Ethical aspects

In this study, exclusively publicly accessible data were used, without individual identification of the participants. The information provided was treated confidentially from its origin and was used exclusively for statistical purposes.

## RESULTS

The "overall fixed cohort" consisted of the 199,999 individuals who had records of all interviews carried out (n=199,999). The "tested fixed cohort" consisted of a subpopulation of the 7,832 individuals from the overall fixed cohort (n=7,832) who underwent monthly testing between July and November (Figure 1).

The frequency of healthcare occupations differed between the cohorts, being higher in the fixed cohort tested (11.8%) when compared to the proportion in the general fixed cohort (1.8%) (Table 1). The proportion of completed higher education in the fixed cohort tested (31.2%) was greater than the proportion of this level of education in the general fixed cohort (12.7%), and the greatest age difference occurred in the range between 30 and 39 years old in the fixed cohort tested (27.7%), when compared to the proportion in this age group in the general fixed cohort (16.2%).

**Table 1:** Sociodemographic characterization of the baselines of the general fixed cohort (n=199,999) and the fixed cohort tested (n=7,832). Brazil, May/2020.

Characteristics		Overall fixed cohort			Fixed cohort tested*		
		n	%	(95%CI)	n	%	(95%CI)
Region							
	Southeast	70,529	47.2	(46.5–47.9)	2,701	47.8	(46.1–49.5)
	Northeast	55,064	24.9	(24.4–25.5)	2,451	25.7	(24.6–26.9)
	South	36,586	14.5	(14.1–14.9)	1,009	**10.6**	**(9.9–11.4)**
	Central West	17,846	6.4	(6.1–6.8)	687	6.8	(5.9–7.9)
	North	19,974	6.9	(6.7–7.2)	984	**9.1**	**(8.4–9.8)**
Age range, in years							
	0–9	21,873	13.9	(13.6–14.1)	156	**2.6**	**(2.1–3.3)**
	10–19	27,012	14.5	(14.3–14.7)	415	**5.5**	**(4.8–6.1)**
	20–29	25,137	16.2	(15.9–16.4)	1,124	**18.0**	**(16.8–19.3)**
	30–39	29,441	16.2	(15.9–16.4)	1,911	**27.7**	**(26.2–29.2)**
	40–49	29,752	13.8	(13.6–14.0)	1,723	**21.0**	**(19.8–22.2)**
	50–59	28,420	11.3	(11.1–11.5)	1,374	**14.3**	**(13.3–15.3)**
	60–69	21,698	7.9	(7.7–8.1)	737	**6.8**	**(6.2–7.5)**
	70–79	11,520	4.3	(4.2–4.4)	276	**3.0**	**(2.5–3.5)**
	≥80	5,146	2.0	(2.0–2.1)	116	**1.2**	**(0.9–1.5)**
Gender							
	Female	104,764	51.1	(50.9–51.3)	4,233	52.3	(51.0–53.5)
	Male	95,235	48.9	(48.7–49.1)	3,599	47.7	(46.5–49.0)
Race or skin color							
	White	89,567	46.0	(45.4–46.6)	3,655	**49.3**	**(47.5–51.2)**
	Black	16,215	8.7	(8.4–9.0)	673	9.4	(8.4–10.4)
	Yellow	1,259	0.7	(0.7–0.8)	47	0.6	(0.4–0.9)
	Brown	92,235	44.2	(43.7–44.8)	3,424	**40.3**	**(38.6–42.0)**
	Indigenous	679	0.3	(0.2–0.3)	30	0.3	(0.2–0.5)
	Not declared	44	<0.1	(<0.1–<0.1)	3	<0.1	(<0.1–<0.1)
Residence area							
	Rural	45,651	86.0	(85.6–86.4)	803	**93.8**	**(93.3–94.4)**
	Urban	154,348	14.0	(13.6–14.4)	7,029	**6.2**	**(5.6–6.7)**
Education (of people aged 25 years old or older)							
	Completed higher education or more	26,065	12.7	(12.3–13.0)	2,453	**31.2**	**(29.7–32.8)**
	Complete secondary education	44,552	23.0	(22.7–23.3)	2,606	**34.3**	**(32.9–35.8)**
	Complete primary education	21,068	10.2	(10.0–10.4)	704	**9.0**	**(8.1–10.0)**
	Incomplete primary education or less	49,200	19.0	(18.7–19.4)	1,147	**11.6**	**(10.7–12.6)**
	Not applicable	59,114	35.1	(34.8–35.4)	922	**13.8**	**(12.8–14.9)**
Work (of people aged 14 years old or older)							
	Business	8,660	4.7	(4.5–4.8)	415	5.4	(4.7–6.2)
	Health	3,477	1.8	(1.7–1.8)	903	**11.8**	**(10.8–12.9)**
	Transport	4,072	2.2	(2.1–2.2)	241	**3.2**	**(2.8–3.8)**
	Food	2,047	1.1	(1.0–1.2)	101	1.4	(1.1–1.9)
	Other higher education professions	11,112	5.6	(5.4–5.8)	939	**12.0**	**(11.0–13.1)**
	Industry	11,659	6.4	(6.2–6.6)	500	**7.7**	**(6.9–8.7)**
	Agriculture	11,057	3.5	(3.4–3.7)	172	**1.5**	**(1.2–1.8)**
	Other services	27,493	14.8	(14.5–15.0)	1,974	**26.6**	**(25.2–28.1)**
	Not declared	88,150	40.5	(40.2–40.9)	2,326	**26.3**	**(25.0–27.7)**
	Not applicable	32,272	19.5	(19.2–19.7)	261	**4.0**	**(3.3–4.8)**

n: Unweighted frequency of observations; %, Proportion of weighted column, referring to the total population estimated for the overall fixed cohort (N_estim_.=210,869,401) and for the fixed cohort tested (N_estim_.=8,332,292); 95%CI: 95% confidence interval for the proportion taking into account sample weighting.

* The proportions and 95% confidence intervals highlighted in bold represent statistically significant differences between the proportions of the fixed cohort tested and their analogues in the overall fixed cohort.

**Figure 2:**
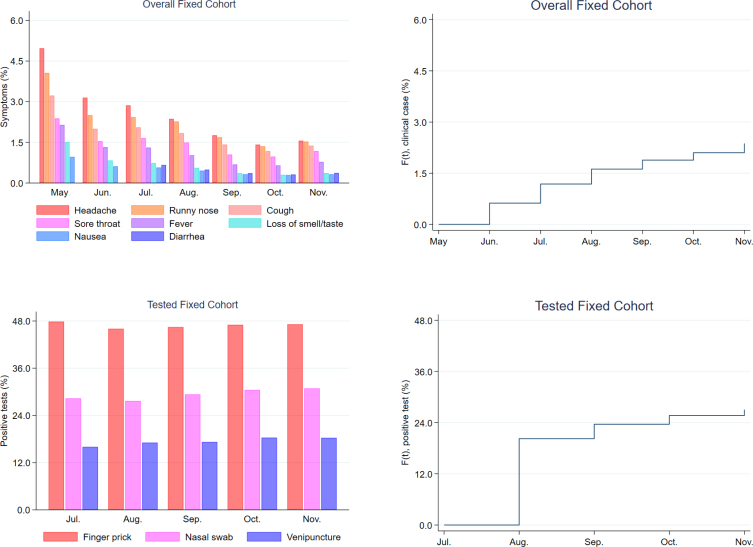
Prevalence of symptoms and cumulative probability of incidence of COVID-19 cases in the general fixed cohort and proportion of positivity for Sars-CoV-2 and cumulative probability of incidence of positive tests in the fixed cohort tested, according to the month of reference. Brazil, 2020.

The prevalence of signs and symptoms related to COVID-19 reduces over the reference months (Figure 2). The most frequent symptom is headache, and its prevalence decreased between May (5.0%) and November (1.6%) 2020. In the fixed cohort tested, the proportions of positive tests collected through nasal swabs increased between July (28.3%) and November (30.9%). The proportions of positive tests collected by venipuncture increased between July (16.0%) and November (18.3%); and the proportion of positive tests collected by finger prick increased in the period between July (47.8%) and November (47.2%).

Notes:

The question regarding the symptom "diarrhea" was implemented from July onward, as were all questions about testing.

The case confirmed by clinical criteria of covid-19 in the overall fixed cohort was determined by the presence of flu-like syndrome associated with loss of smell or taste, in accordance with the recommendations of the Brazilian Ministry of Health.

Positive testing for Sars-CoV-2 in the fixed cohort tested was determined by reporting a positive result in tests collected via finger prick, nasal swab, or venipuncture.

All values take sampling weight into account.

F(t), function of accumulated probability of the occurrence of failures, estimated by the non-parametric Kaplan-Meier statistic.

Based on the accumulated probability function of the occurrence of failures (Figure 2), it appears that, during a period of six months of follow-up, new apparent cases of COVID-19 occurred in 2.4% of individuals who had not previously presented the combination of symptoms, in the overall fixed cohort. In the fixed cohort tested, the cumulative probability of incidence of positive tests was equivalent to 27.1% of individuals without previous positive results.

All studied variables were taken into account in the adjusted models, in order to isolate the magnitude of the association between exposures and outcomes from potentially confounding variables (Table 2). In view of this, it appears that the group of Brazilians whose highest level of education was represented by complete primary education had a 22% higher risk of being considered a symptomatic case of COVID-19, when compared to the group composed of Brazilians with complete higher education, between May and November 2020, regardless of the time and region of residence, age range, gender, area of residence, and work (HR=1.22, 95%CI 1.03–1.44). Among individuals with monthly testing frequency, it appears that healthcare professionals had a 77% higher risk of presenting positive tests when compared to commercial workers (HR=1.77, 95%CI 1.19–2.64).

**Table 2:** Estimated event count, incidence density, and hazard ratio for the occurrence of COVID-19 cases in the overall fixed cohort and positive Sars-CoV-2 tests in the fixed tested cohort, according to sociodemographic aspects. Brazil, 2020.

Characteristics		Case*				Positive test†			
		Estimated number of events‡	Incidence density (per 1,000 person-month)^§^	unadjusted HR (95%CI)//	adjusted HR (95%CI)¶	Estimated number of events‡	Incidence density (per 1,000 person-month)§	unadjusted HR (95%CI)//	adjusted HR (95%CI)¶
Region									
	Southeast	1,936,518	3.30	1 (ref.)	1 (ref.)	504,702	81.38	1 (ref.)	1 (ref.)
	Northeast	1,386,595	4.56	1.38 (1.21–1.57)	1.47 (1.29–1.68)	239,761	79.05	0.97 (0.77–1.21)	0.93 (0.74–1.17)
	South	617,021	3.40	1.03 (0.89–1.2)	1.09 (0.94–1.28)	107,649	77.71	0.96 (0.74–1.24)	0.91 (0.7–1.18)
	Central West	491,692	6.58	1.99 (1.67–2.37)	2.20 (1.84–2.62)	78,663	111.32	1.29 (0.92–1.81)	1.32 (0.95–1.84)
	North	509,362	5.92	1.79 (1.54–2.08)	1.80 (1.55–2.09)	89,325	79.15	0.97 (0.75–1.26)	0.98 (0.76–1.27)
Stage of life									
	Aged	508,756	2.82	1 (ref.)	1 (ref.)	96,938	64.40	1 (ref.)	1 (ref.)
	Middle-aged	1,577,973	5.11	1.81 (1.61–2.03)	1.67 (1.47–1.89)	341,582	80.58	1.22 (0.95–1.55)	1.24 (0.95–1.61)
	Young adult	2,169,513	5.50	1.94 (1.73–2.17)	1.83 (1.60–2.09)	523,464	92.60	1.37 (1.07–1.77)	1.49 (1.11–2.00)
	Adolescent	543,610	3.01	1.07 (0.91–1.25)	1.65 (1.29–2.11)	32,911	46.41	0.73 (0.46–1.15)	0.94 (0.49–1.78)
	Child	141,338	0.84	0.29 (0.23–0.38)	0.78 (0.53–1.14)	25,206	72.19	1.08 (0.57–2.03)	1.16 (0.38–3.53)
Gender									
	Male	2,027,863	3.35	1 (ref.)	1 (ref.)	480,551	81.96	1 (ref.)	1 (ref.)
	Female	2,913,327	4.64	1.38 (1.29–1.48)	1.35 (1.25–1.45)	539,549	81.84	1.00 (0.87–1.15)	0.96 (0.83–1.10)
Race or skin color									
	White	2,016,966	3.53	1 (ref.)	1 (ref.)	529,962	81.66	1 (ref.)	1 (ref.)
	Brown	2,351,543	4.34	1.23 (1.12–1.35)	1.07 (0.97–1.18)	389,233	81.41	0.99 (0.83–1.19)	0.55 (0.17–1.81)
	Black	519,712	4.88	1.38 (1.19–1.61)	1.18 (1.01–1.38)	95,425	94.51	1.13 (0.82–1.54)	1.03 (0.76–1.38)
	Yellow	30,730	3.33	0.94 (0.57; 1.56)	0.86 (0.52; 1.43)	4,892	44.69	0.57 (0.18–1.82)	0.55 (0.17–1.81)
	Indigenous	21,635	6.59	1.86 (1.13–3.07)	1.4 (0.84–2.31)	587	9.01	,,,	,,,
Residence area									
	Rural	524,516	3.02	1 (ref.)	1 (ref.)	46,932	69.72	1 (ref.)	1 (ref.)
	Urban	4,416,674	4.17	1.38 (1.2–1.59)	1.38 (1.2–1.6)	973,168	82.60	1.17 (0.85–1.62)	1.26 (0.88–1.81)
Education (of people aged 25 years old or older)									
	Completed higher education or more	769,776	4.92	1 (ref.)	1 (ref.)	355,507	78.07	1 (ref.)	1 (ref.)
	Complete secondary education	1,513,699	5.40	1.10 (0.97–1.24)	1.15 (1.00–1.32)	361,675	94.99	1.18 (0.98–1.42)	1.32 (1.09–1.61)
	Complete primary education	624,464	5.00	1.02 (0.87–1.19)	1.22 (1.03–1.44)	85,611	84.84	1.06 (0.75–1.51)	1.26 (0.89–1.78)
	Incomplete primary education or less	955,004	4.08	0.83 (0.72–0.95)	1.13 (0.96–1.32)	103,326	83.16	1.04 (0.81–1.35)	1.37 (1.02–1.83)
Work (of people aged 14 years old or older)									
	Business	304,612	4.60	1 (ref.)	1 (ref.)	53,009	74.33	1 (ref.)	1 (ref.)
	Health	165,203	6.22	1.46 (1.11–1.92)	1.42 (1.08–1.87)	154,737	127.45	1.60 (1.09–2.35)	1.77 (1.19–2.64)
	Transport	120,816	3.66	0.86 (0.65–1.12)	0.98 (0.75–1.29)	30,165	82.42	1.09 (0.62–1.9)	1.03 (0.59–1.8)
	Food	63,718	3.64	0.91 (0.66–1.25)	0.9 (0.65–1.24)	20,011	181.39	2.07 (1.13–3.81)	1.99 (1.11–3.58)
	Other higher education professions	357,585	4.51	0.97 (0.79–1.20)	1.1 (0.88–1.37)	146,823	83.03	1.11 (0.75–1.63)	1.26 (0.84–1.91)
	Industry	348,516	3.83	0.83 (0.68–1.01)	0.92 (0.75–1.13)	68,016	81.08	1.08 (0.67–1.72)	1.01 (0.64–1.61)
	Agriculture	102,627	1.87	0.44 (0.33–0.57)	0.56 (0.43–0.74)	16,387	101.49	1.29 (0.47–3.56)	1.34 (0.51–3.52)
	Other services	1,075,569	5.47	1.12 (0.94–1.33)	1.14 (0.96–1.35)	242,537	70.79	0.96 (0.67–1.37)	0.94 (0.66–1.35)
	Not declared	2,128,377	3.99	0.80 (0.68–0.94)	0.91 (0.77–1.08)	255,415	76.07	1.02 (0.7–1.47)	1.15 (0.79–1.68)

Notes: The results from the "Not applicable" category of the education and work variables and the "Not declared" category of the race or skin color variable were omitted due to their low precision. Results from the "Indigenous" category in the fixed cohort test were omitted due to the small sample size.

* Case confirmed by clinical criteria of covid-19 in the overall fixed cohort, determined by the presence of flu-like syndrome associated with loss of smell or taste;

† Positive test for Sars-CoV-2 in the fixed cohort tested, determined by reporting a positive result in tests collected via finger prick, nasal swab, or venipuncture;

‡ Estimated number of events in the overall fixed cohort, from June to November 2020 (N_estim_.=210,869,401) and in the fixed cohort tested, from August to November 2020 (N_estim_.=8,332,292);

§ Unadjusted incidence density, resulting from the division between the estimated number of first events and the number of months contributed by individuals in each group (person-month), multiplied by 1,000;

// Hazard ratio (HR) and 95% confidence interval (95%CI), obtained by simple Cox regression;

¶ Hazard ratio (HR) and 95% confidence interval (95%CI), obtained by Cox regression adjusted for all sociodemographic variables presented.

## DISCUSSION

The overall and tested fixed cohorts differed in relation to educational level, age range, and occupation category, indicating that frequent access to testing was unequal between population strata. The incidence rate of symptomatic cases differed between categories of geographic region, gender, stage of life, area of residence, race or skin color and education, while viral detection differed according to work, education, and age range.

Limitations of this study include the percentage of households in the Pnad sample that did not have a telephone, the proportion of interviews that were not paired due to insufficient information on day, month, and year of birth, and the fact that information on tests for COVID-19 were only included from July 2020 onward. These limitations were, in part, reduced by the use of sample weighting, given the application of post-stratification according to gender, age, and education, potentially increasing the representativeness of individuals who did not have their birth dates informed or who did not have telephone devices^16,17,19^.

Regarding testing, according to official data, it appears that during epidemiological week 30 of 2020 (July 19–25^th^) only 1,624 tests were carried out across Brazil, with the average number of tests between epidemiological weeks 30 and 50 (July 19^th^–December 12^th^) was equivalent to 166,678 tests per week^22^. Therefore, the absence of questions about testing in Pnad before July seems consistent with the situation of low testing in Brazil. Despite efforts to increase testing capacity in the country, it was found that there was a shortage of tests and reagents, resulting from the lack of coordination and anticipation of reagent purchases by the government, as well as fragmentation in financing and distribution of tests^23^.

According to our estimates, the highest relative risk of COVID-19 cases occurred in the Central West region, followed by the North and Northeast, when compared to the Southeast region. This order was similar to that observed in official notifications until epidemiological week 50 of 2020 (December 6–12^th^), in which higher incidence coefficients were reported for the Central West region, followed by the North, Northeast, South, and Southeast regions^22^.

The female group presented a higher risk of cases according to the combination of self-reported symptoms, but not according to positivity. Compared to men, women are more attentive to their self-care^24^ and have a worse self-assessment of their health status^25^. Thus, the female group may have had individuals who were more attentive to their symptoms, reporting them more reliably. Lower incidences were observed in aged people, consistent with the massive number of campaigns aimed at preventing contagion in this age range^26^.

Among individuals who reported having black skin color, there was a greater risk of clinical cases, but not positive tests. Greater occurrence of the combination of symptoms that represents a clinical case can be explained by material inequalities, related to precarious housing conditions and high housing density^27^. Furthermore, underlying health conditions that are more prevalent in this population may have influenced the severity and, consequently, the perception of the symptoms of COVID-19^28^. The lack of association in positivity for Sars-CoV-2 is related to the lack of distinctions between the molecular mechanisms of virus action between race groups^29^.

Between May and November 2020, there was a greater risk of apparent clinical cases of COVID-19 among Brazilians in the Central West, North, and Northeast regions, especially among women, residents in urban areas, people with education up to elementary school and high school, self-declared as black, and health workers. According to the occurrence of positive tests for Sars-CoV-2, between July and November 2020, there was a greater risk in groups made up of young adults, with no education and with complete secondary education and with occupations related to health and food. By expanding the scope of Pnad, the potential of reusing surveys for national epidemiological intelligence was demonstrated, given the richness of Brazil's public data ecosystem.

## Supplementary Material


