# Supplementary material for: Sociodemographic inequalities in the incidence of COVID-19 in National Household Sample Survey cohort, Brazil, 2020
Source: Rev Bras Epidemiol. 2024 Mar 18;27:e240012. doi: 10.1590/1980-549720240012 (PMC10946290; doi:10.1590/1980-549720240012)
Supplement: Supplementary file 1 [file 1980-5497-rbepid-27-e240012-Suppl01.docx]

**MATERIAL SUPLEMENTAR**

**Título do manuscrito:** Desigualdades sociodemográficas na incidência de COVID-19 em coorte da Pesquisa Nacional por Amostra de Domicílios, Brasil, 2020.

**Imagem suplementar 1 – Distribuição etária da amostra da coorte fixa geral (n=199.999) e da coorte fixa testada (n=7.832), de acordo com o sexo. Brasil, maio/2020.**

Nota: Frequências não ponderadas.

**Tabela suplementar 1 – Prevalência de sintomas de COVID-19 e proporção de testes positivos para SARS-CoV-2, de acordo com o mês. Brasil, 2020.**

| **Sintomas ou tipo do teste** | **Mês** | | | | | | |
| --- | --- | --- | --- | --- | --- | --- | --- |
|  | **Maio** | **Jun.** | **Jul.** | **Ago.** | **Set.** | **Out.** | **Nov.** |
| **Coorte fixa geral** | **Prevalência de sintomas**  % (IC_95%_) | | | | | | |
| Dor de cabeça | 5,0 (4,8; 5,1) | 3,1 (3,0; 3,3) | 2,9 (2,7; 3,0) | 2,4 (2,3; 2,5) | 1,8 (1,7; 1,9) | 1,4 (1,3; 1,5) | 1,6 (1,5; 1,6) |
| Coriza | 4,1 (3,9; 4,2) | 2,5 (2,4; 2,6) | 2,4 (2,3; 2,5) | 2,3 (2,2; 2,4) | 1,7 (1,6; 1,8) | 1,4 (1,3; 1,4) | 1,5 (1,4; 1,6) |
| Tosse | 3,2 (3,1; 3,4) | 2,0 (1,9; 2,1) | 2,1 (2,0; 2,2) | 1,8 (1,7; 1,9) | 1,4 (1,3; 1,5) | 1,2 (1,1; 1,3) | 1,4 (1,3; 1,5) |
| Dor de garganta | 2,4 (2,3; 2,5) | 1,5 (1,5; 1,6) | 1,7 (1,6; 1,8) | 1,5 (1,4; 1,6) | 1,0 (1,0; 1,1) | 1,0 (1,0; 1,1) | 1,2 (1,1; 1,3) |
| Febre | 2,1 (2,0; 2,3) | 1,3 (1,2; 1,4) | 1,3 (1,2; 1,4) | 1,0 (1,0; 1,1) | 0,7 (0,6; 0,7) | 0,6 (0,6; 0,7) | 0,8 (0,7; 0,8) |
| Perda de olfato/ paladar | 1,5 (1,4; 1,6) | 0,8 (0,8; 0,9) | 0,7 (0,7; 0,8) | 0,6 (0,5; 0,6) | 0,4 (0,3; 0,4) | 0,3 (0,3; 0,3) | 0,4 (0,3; 0,4) |
| Náusea | 1,0 (0,9; 1,0) | 0,6 (0,6; 0,7) | 0,6 (0,5; 0,6) | 0,5 (0,4; 0,5) | 0,3 (0,3; 0,4) | 0,3 (0,3; 0,3) | 0,3 (0,3; 0,4) |
| Diarreia | – | – | 0,7 (0,6; 0,7) | 0,5 (0,4; 0,5) | 0,4 (0,3; 0,4) | 0,3 (0,3; 0,4) | 0,4 (0,3; 0,4) |
| **Coorte fixa testada** | **Proporção de positividade**  % (IC_95%_) | | | | | | |
| Punção digital | **–** | **–** | 47,8 (45,8; 49,8) | 46,0 (44,2; 47,9) | 46,5 (44,6; 48,3) | 47,0 (45,2; 48,9) | 47,2 (45,3; 49,0) |
| *Swab* nasal | **–** | **–** | 28,3 (25,4; 31,5) | 27,7 (24,9; 30,7) | 29,4 (26,7; 32,1) | 30,5 (27,9; 33,2) | 30,9 (28,3; 33,6) |
| Punção venosa | **–** | **–** | 16,0 (14,4; 17,8) | 17,1 (15,4; 19,0) | 17,3 (15,6; 19,1) | 18,3 (16,6; 20,2) | 18,3 (16,5; 20,2) |

Nota: Percentuais ponderados, levando em consideração o desenho amostral. A questão referente ao sintoma “diarreia” foi inserida a partir do mês de julho, assim como todas as questões sobre testagem.

**Imagem suplementar 2 – Probabilidade acumulada de incidência de casos de COVID-19 e de testes positivos para SARS-CoV-2, segundo estratos socioeconômicos e demográficos, de acordo com a coorte estudada. Brasil, 2020.**


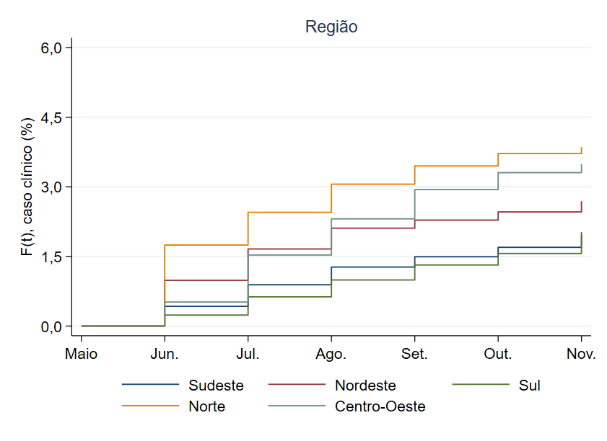


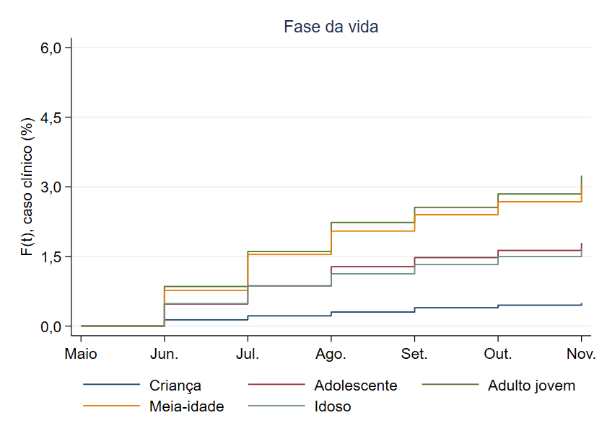

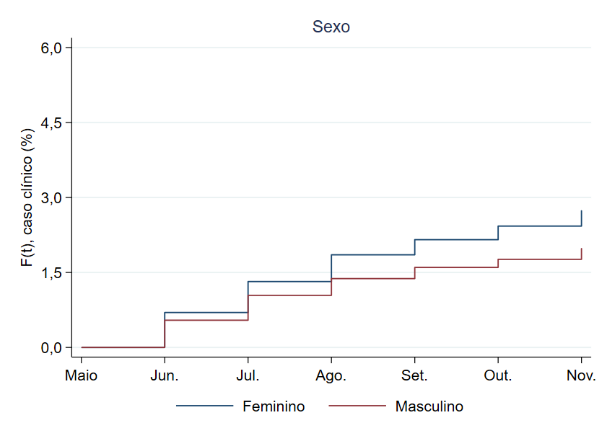

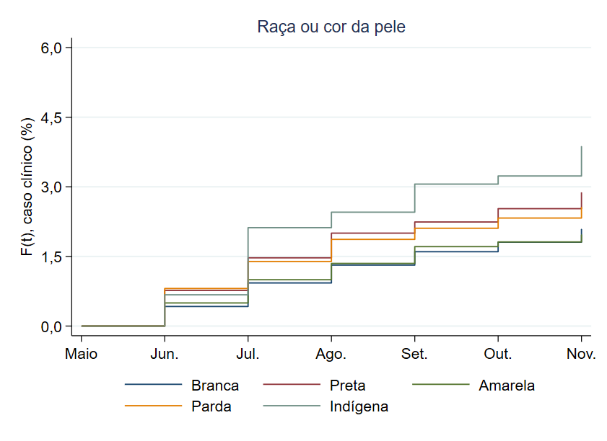

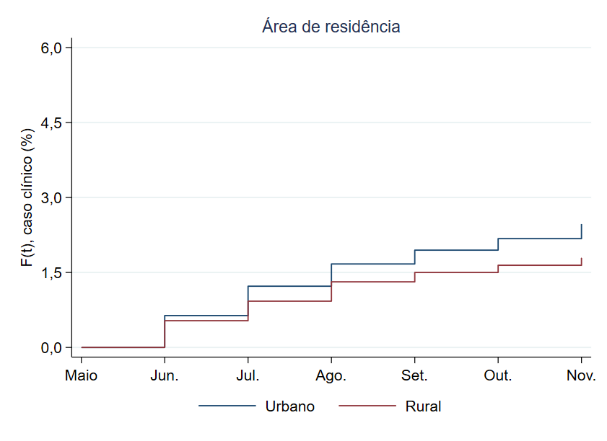

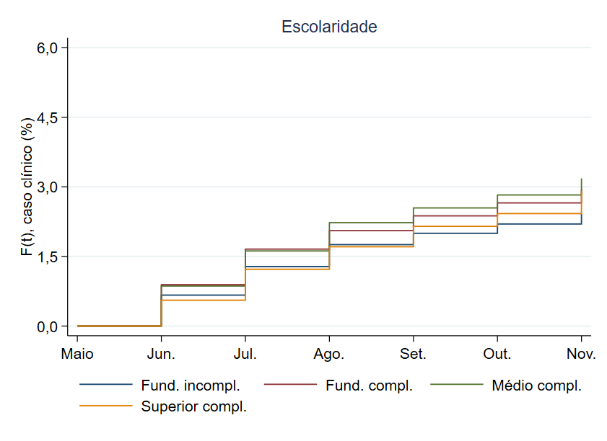

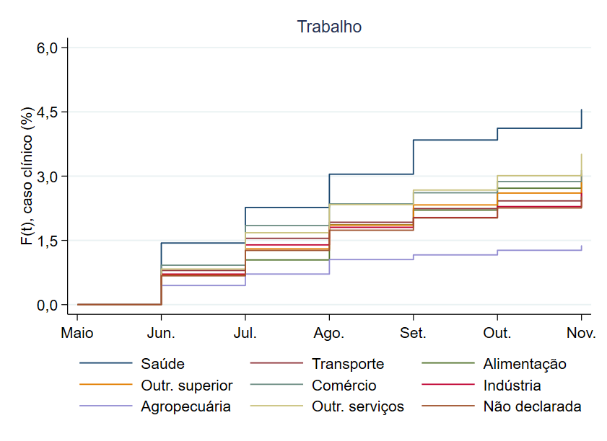


**______________________________________________________________________**


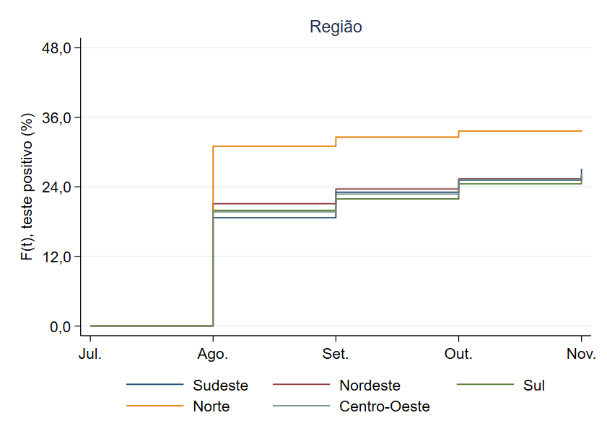


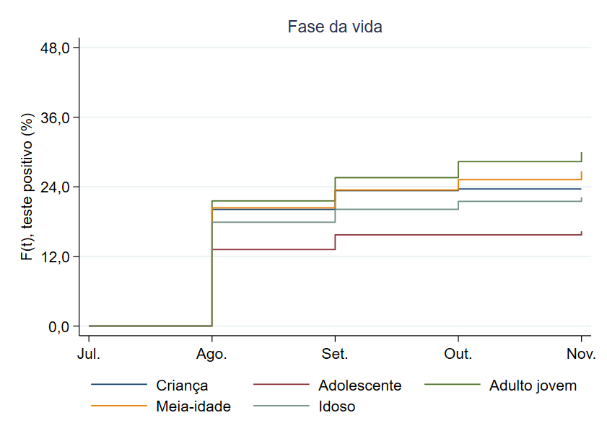

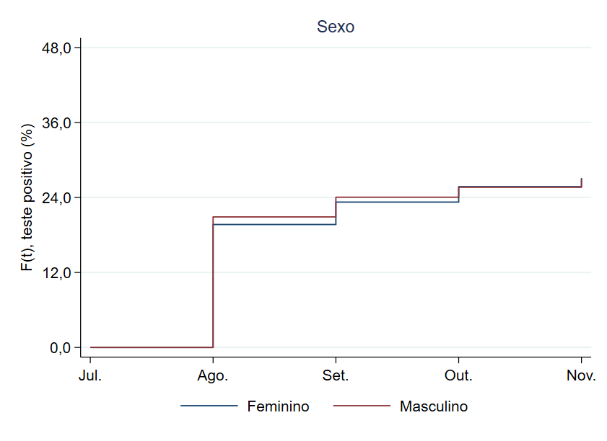

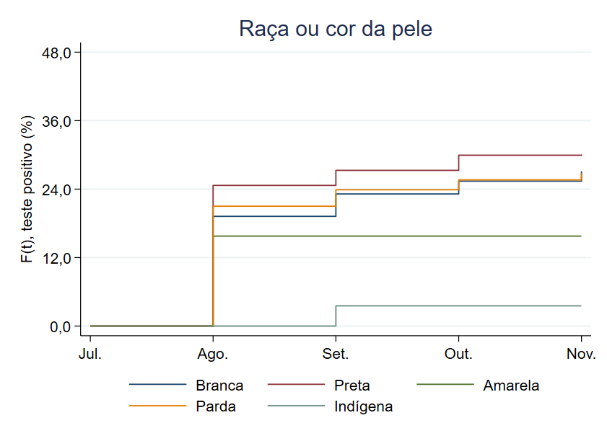

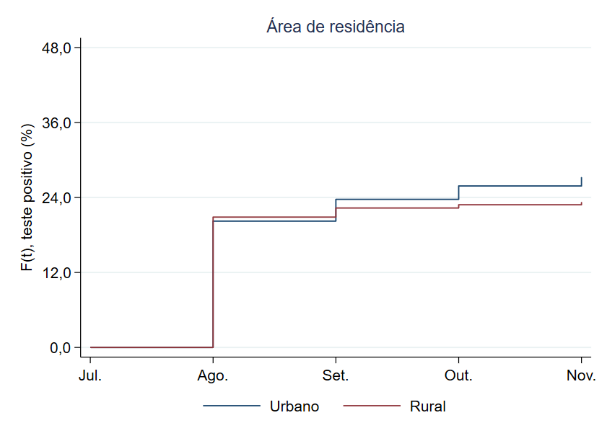

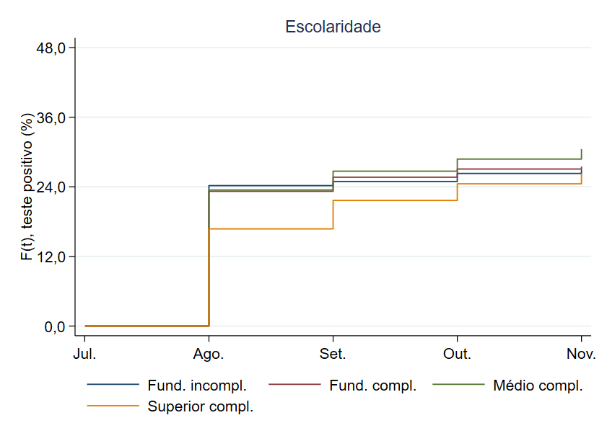

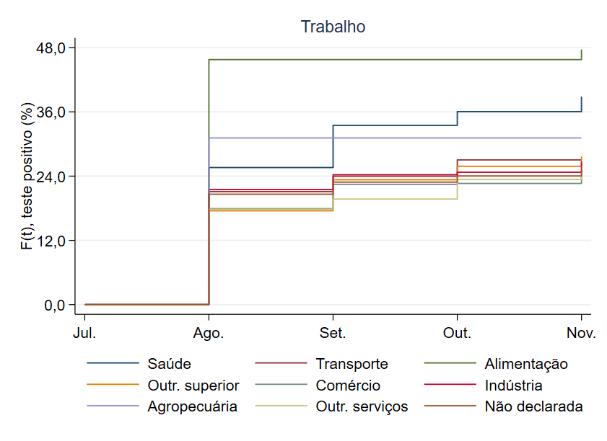


Nota:

F(t), função de probabilidade acumulada da ocorrência de falhas, estimada pela estatística não-paramétrica de Kaplan-Meier. Os valores levam em consideração a ponderação amostral.

**Quadro suplementar 1 – Composição da variável referente ao trabalho das pessoas de 14 anos ou mais. Brasil, maio/2020.**

| **Categoria e respectivos cargos ou funções** |
| --- |
| **Comércio** |
| Operador de Telemarketing |
| Comerciante (dono do bar, da loja etc.) |
| Balconista, vendedor de loja |
| Vendedor a domicílio, representante de vendas, vendedor de catálogo (Avon, Natura etc.) |
| Vendedor ambulante (feirante, camelô, comerciante de rua, quiosque) |
| **Saúde** |
| Médico, enfermeiro, profissionais de saúde de nível superior |
| Técnico, profissional da saúde de nível médio |
| Cuidador de crianças, doentes ou idosos |
| **Transporte** |
| Motorista (de aplicativo, de taxi, de van, de mototáxi, de ônibus) |
| Motorista de caminhão (caminhoneiro), |
| Motoboy, |
| Entregador de mercadorias (de restaurante, de farmácia, de loja, Uber Eats, IFood, Rappy etc.) |
| **Alimentação** |
| Cozinheiro e garçon (de restaurantes, empresas) |
| Padeiro, açougueiro e doceiro |
| **Out. superior** |
| Professor da educação infantil, de ensino fundamental, médio ou superior, |
| Pedagogo, professor de idiomas, música, arte e reforço escolar |
| Policial civil |
| Artista, religioso (padre, pastor etc.) |
| Diretor, gerente, cargo político ou comissionado |
| **Indústria** |
| Pedreiro, servente de pedreiro, pintor, eletricista, marceneiro |
| Mecânico de veículos, máquinas industriais etc. |
| Artesão, costureiro e sapateiro |
| Cabeleireiro, manicure e afins |
| Operador de máquinas, montador na indústria; |
| **Agropecuária** |
| Agricultor, criador de animais, pescador, silvicultor e jardineiro |
| Auxiliar da agropecuária (colhedor de frutas, boia fria, etc.) |
| **Out. serviços** |
| Empregado doméstico, diarista, cozinheiro (em domicílios particulares), |
| Faxineiro, auxiliar de limpeza etc. (em empresa pública ou privada), |
| Auxiliar de escritório, escriturário |
| Secretária, recepcionista |
| Cabeleireiro, manicure e afins |
| Segurança, vigilante, outro trabalhador dos serviços de proteção |
| Porteiro, zelador |
| Artista, religioso (padre, pastor etc.) |
| Outro técnico ou profissional de nível médio |
| Outros |
